# Structural analysis of human glycoprotein butyrylcholinesterase using atomistic molecular dynamics: The importance of glycosylation site ASN_241_

**DOI:** 10.1371/journal.pone.0187994

**Published:** 2017-11-30

**Authors:** Austen Bernardi, Karl N. Kirschner, Roland Faller

**Affiliations:** 1 Department of Chemical Engineering, University of California–Davis, Davis, California, United States of America; 2 Bonn–Rhein–Sieg University of Applied Sciences, Sankt Augustin, Germany; Weizmann Institute of Science, ISRAEL

## Abstract

Human butyrylcholinesterase (BChE) is a glycoprotein capable of bioscavenging toxic compounds such as organophosphorus (OP) nerve agents. For commercial production of BChE, it is practical to synthesize BChE in non–human expression systems, such as plants or animals. However, the glycosylation profile in these systems is significantly different from the human glycosylation profile, which could result in changes in BChE’s structure and function. From our investigation, we found that the glycan attached to ASN_241_ is both structurally and functionally important due to its close proximity to the BChE tetramerization domain and the active site gorge. To investigate the effects of populating glycosylation site ASN_241_, monomeric human BChE glycoforms were simulated with and without site ASN_241_ glycosylated. Our simulations indicate that the structure and function of human BChE are significantly affected by the absence of glycan 241.

## Introduction

Butyrylcholinesterase (BChE) is a prophylactic therapeutic glycoprotein for organophosphorus (OP) nerve agents [1]. OP nerve agents are toxic because they inhibit acetylcholinesterase (AChE) [2], the hydrolyzing enzyme of neurotransmitter acetylcholine [3]. Some examples of OP nerve agents include sarin, soman and tabun. BChE must be administered within two minutes of an OP exposure in humans, which inactivates the OP before local AChE is affected [4]. For a comprehensive review of OP nerve agents and their treatment, the reader is referred to reference [5].

BChE naturally exists in humans and animals in monomeric, dimeric and tetrameric forms, where each monomer acts as a stoichiometric scavenger of an OP nerve agent. These oligomeric forms of BChE may be in either soluble, globular forms or anchored to a membrane [6]. Monomeric BChE contains 574 amino acids with nine asparagine (N)–linked glycans at residue indices 17, 57, 106, 241, 256, 341, 455, 481 and 486 [7]. The BChE N–glycosylation sites follow the standard triplet motif: asparagine–*X*–threonine/serine, where *X* is any amino acid except proline [8]. N–glycosylation is a heterogeneous process that can affect pharmacokinetic stability, immunocompatibility, reactivity, and thermal and kinetic stability [9]. Glycans influence these factors through two motifs: direct interaction with their covalently linked protein, or through external interactions with other molecules.

Structurally, BChE contains two distinct domains: the core domain (amino acids 1–529), and the tetramerization domain (amino acids 530–574). The core domain is the stable folded portion of monomeric BChE, and houses a single active gorge that contains the catalytic SER_198_ residue at its base. BChE activity is irreversibly inhibited by OP nerve agents through covalent modification of SER_198_. This reaction simultaneously deactivates BChE and destroys the OP nerve agent, explaining BChE’s bioscavenging ability [10]. However, there is evidence that OP nerve agents are capable of covalently binding protein residues other than serine, such as tyrosine and lysine [11]. The tetramerization domain is the flexible portion of BChE, and participates in complexation of the BChE tetramer about a central polyproline helix.

The nine N–glycosylation sites of BChE are spread evenly across the core domain, with the exception of the surfaces that contact other monomers in the tetramer. Of particular interest is glycan 241, which, through the simulation presented herein, was found to interact with both the tetramerization domain and the surface residues of the active site gorge. No other glycans directly interact with the gorge, and the only other glycan to interact with the tetramerization domain is glycan 256. Therefore, glycan 241 is likely the most relevant glycan when considering the functional performance of BChE. In this work we study the effects that glycan 241 has on the structure and dynamics of monomeric human BChE using atomistic molecular dynamics (MD). From the results, we hypothesize that the glycosylation state has a direct impact on BChE’s bioscavenger function. Atomistic MD enables the determination of time–ordered atomic trajectories for timescales on the order of picoseconds to a microsecond and lengthscales on the order of nanometers [12], and is a powerful tool for investigating condensed matter systems in high detail. There have been quite a number of works on the simulation of human BChE, ranging from mutation studies [13] to BChE–cocaine binding simulations [14] to BChE tetramer model building [15]. However, this study is unique in that all of the previous simulation work on BChE was with aglycosylated BChE, whereas this study is the first to include glycosylated simulations of BChE.

Glycans are branched, flexible chains of carbohydrates that explore an ensemble of conformations at equilibrium conditions. This complicates the structural characterization of glycans using laboratory experiments. Conversely, atomistic MD simulations are well suited for glycan characterization since their high–resolution atomic trajectories are easily analyzed to obtain structural information. The main limitation of atomistic MD simulations are their restricted length– and time–scales. Fortunately, improvements in computational power and efficiency continues to progress, enabling an ever–improving characterizations of glycoprotein ensembles.

## Materials and methods

**Fig 1:**
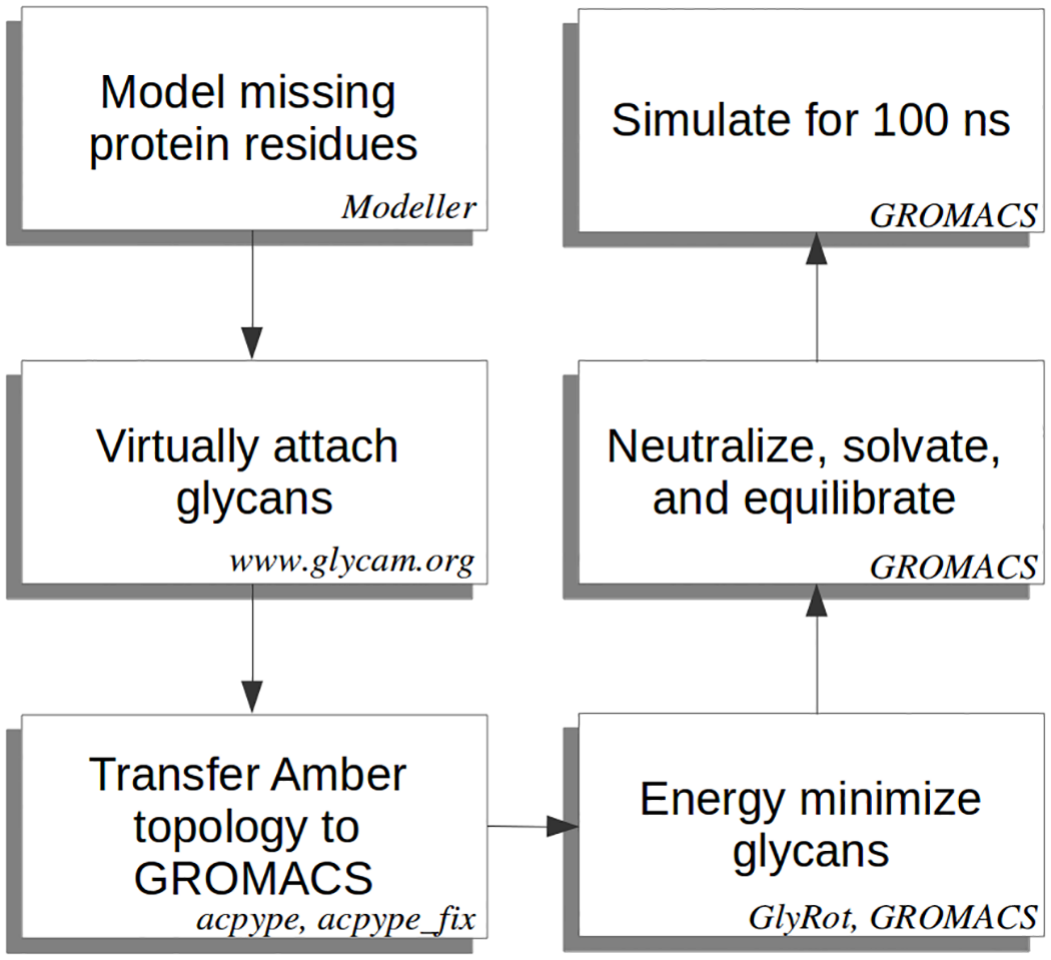
Glycoprotein simulation workflow. Flowchart describing the overall procedure used to simulate glycoproteins.

The overall process to simulate BChE glycoforms is summarized in Fig 1. The sections below describe the process components in detail. All molecular figures were generated using VMD [16] and its built–in STRIDE secondary structure function [17] for protein representation. Cavity volumes were generated with VOIDOO [18] using a grid spacing of 0.3 Å and a probe radius of 1.4 Å, emulating a water probe.

### BChE homology modeling

The atomic coordinates of recombinant BChE (rBChE) have been determined using X–ray crystallography (PDB code 1P0I) [19]. However, this rBChE has mutations N17Q, N455Q, N481Q and N486Q, which deactivate four of the nine glycosylation sites. Additionally, only residues 1–529 out of the 574 total were expressed. These modifications were made to reduce the number of flexible residues that inhibit the crystallization process. To transform rBChE to wild–type BChE, we used Modeller 9.16 [20] to revert the mutated residues and to reintroduce the removed residues.

**Fig 2:**
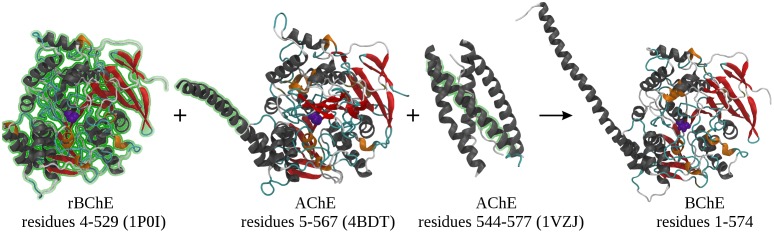
Inputs and results of homology modelling of BChE. Homology model performed using Modeller 9.16 [20]. AChE and BChE are homologically similar, but their residues indices do not map one to one. Only AChE residues within the tetramerization domain (BChE residues 530–574, AChE residues 540–584) were used as model inputs. The active site SER_198_ is shown in purple, with the reader oriented directly above the gorge of the active site. The N terminus is at the right, and the C terminus at the left in all images. Green glow indicates residues used as inputs in the model.

Residues 530–574 of BChE comprise the tetramerization domain, which is a long flexible *α*–helix. AChE is homologically similar to BChE, and has also been studied using X–ray crystallography (PDB code 4BDT) [21]. Performing sequence alignment with blastp [22], we used 4BDT as a template for modelling residues 530–557 of BChE. Similarly, segments of the AChE tetramerization domain complexed with a left–handed polyproline helix have been crystallized (PDB code 1VZJ) [23], and were used as an input for modelling BChE residues 534–567. Residues 568–574 do not have an experimentally crystallized structure and were simply modelled initially as an *α*–helix. All inputs were simultaneously used to generate our BChE homology model. A subsequent 20 ns simulation showed residues 568–574 degenerate to random coil. These 7 residues are in fact intrinsically disordered, as tested with SPOT–Disorder [24]. The results of our homology model and subsequent 20 ns simulation are shown in Fig 2.

### Force fields

All simulations were performed using the GROMACS 5.1 simulation suite [25–27]. Protein atoms were modeled using the Amber ff14SB force field [28], glycan atoms were modeled using the GLYCAM06-j force field [29], and water atoms were modeled using the SPC/E force field [30]. Since the GLYCAM06 force field has not been incorporated into GROMACS yet, Amber topologies were first created using AmberTools16’s LeaP [31], and then exported to GROMACS topologies using a modified version of ACPYPE [32]. We caution the reader that the published ACPYPE is designed to export the base AMBER force fields, which use uniform scaling constants for nonbonded 1–4 interactions and non–negative dihedral force constants. The GLYCAM06-j force field uses variable scaling constants for 1–4 interactions and contains some negative dihedral force constants. Consequently, the published ACPYPE will not properly convert GLYCAM06-j. We modified ACPYPE such that negative dihedral force constants were allowed, and every nonbonded 1–4 interaction was treated separately using the GROMACS [nb_pairs] topology directive. S1 Fig shows the ability of our modified ACPYPE (to be published) versus the published ACPYPE to reproduce a representative dihedral distribution of N–acetylglucosamine from the GLYCAM06-j force field. Our modified version of ACPYPE is publicly available in the GitHub repository https://github.com/austenb28/acpype_fix.

### Virtual glycan attachment

All glycans were virtually attached using the glycoprotein builder of the GLYCAM web portal [33]. Amber topologies were generated and converted to GROMACS topologies using the methodology described above. Once the GROMACS topologies were generated, the attached glycans were sequentially energy minimized in vacuo along the N–glycosidic and *ω*_*p*_ bonds (see S2 Fig), in order to relax the system and remove steric clashes for subsequent atomistic MD simulations. The energy minimization process was coded in C++ using the GROMACS environment, and the two rotatable bonds were each fully rotated at ten degree increments, yielding 36^2^ energy calculations per glycan. Only glycans that were previously minimized in the sequential process were included in the energy calculations.

**Fig 3:**
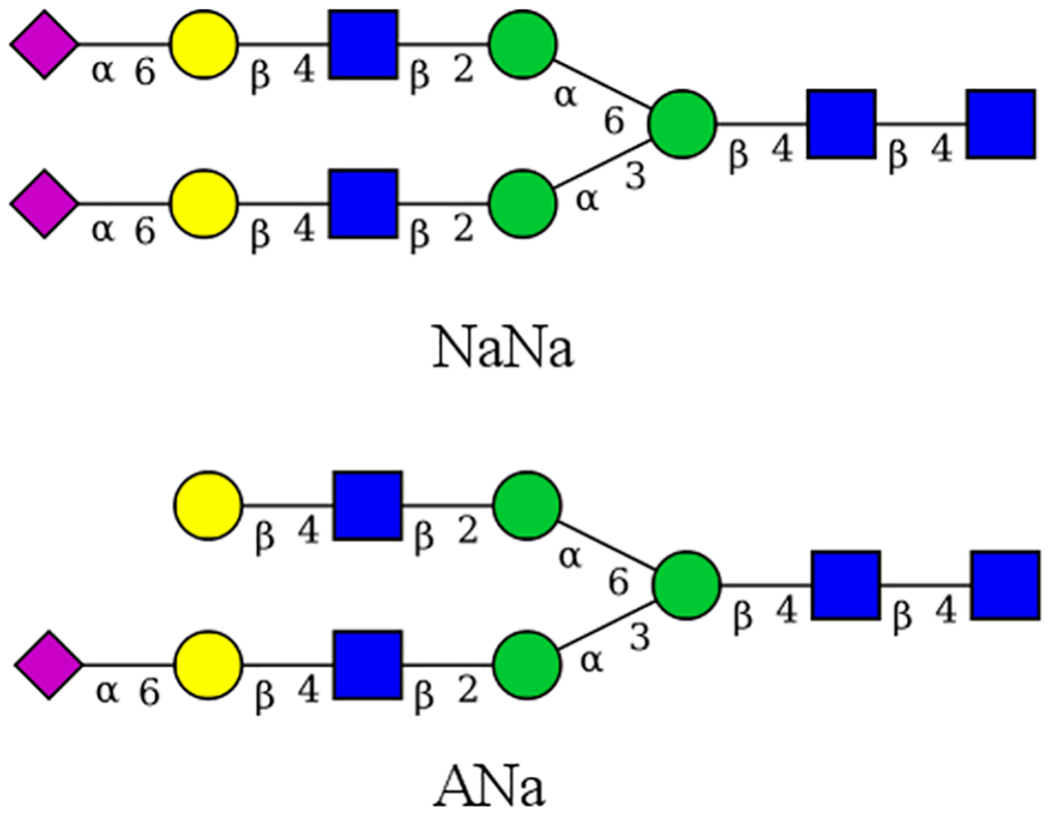
Simulated glycans. CFG cartoon representations of the proglycan abbreviations created with GlycanBuilder [35]. Blue squares are N–acetylglucosamine, green circles are mannose, yellow circles are galactose, and magenta diamonds are N-Acetylneuraminic acid. Linkages and anomeric centers are labeled.

Table 1 details the BChE glycoforms that were simulated, while Fig 3 shows the CFG cartoon representations of the glycans (i.e. NaNa and ANa) present in our simulations. The human glycosylation profile of BChE was taken from the most predominant forms of reference [34]. NaNa represents complex, biantennary human glycans, while ANa represents complex, biantennary *α* 1,3–arm monosialylated human glycans. The glycan 241 (–) glycoform differs from the human glycoform in that it is aglycosylated at site ASN_241_. A third simulation was conducted that is identical to the human glycoform, but whose starting structure was taken from the final coordinates of the glycan 241 (–) simulation. This simulation will be specified separately as glycan 241 (+). As done above, the newly attached glycan 241 was minimized prior to MD simulation.

**Table 1:** Simulated BChE glycoforms and their corresponding glycan distribution.

Glycan site	Glycan 241 (–)	Human and Glycan 241 (+)
N17	ANa	ANa
N57	NaNa	NaNa
N106	ANa	ANa
N241	–	NaNa
N256	ANa	ANa
N341	NaNa	NaNa
N455	NaNa	NaNa
N481	NaNa	NaNa
N486	NaNa	NaNa

Proglycan nomenclature from www.proglycan.com. The ‘–’ symbol indicates the absence of a glycan. Note that A = A^4^ and Na = Na^6–4^. Human and glycan 241 (+) simulations differ by their starting conformations.

### General simulation setup

BChE histidine protonation states were determined using Reduce [36]. All non–water bonds were constrained using LINCS [37], while water bonds and angles were constrained using the analytical SETTLE method [38]. Two initial model systems were created—one where the glycan 241 was present (i.e. human) and one where glycan 241 was absent (i.e. glycan 241 (–)). The periodic, cubic simulation boxes for the human and glycan 241 (–) glycoforms had initial dimensions of 16.1 × 16.1 × 16.1 nm^3^ and 15.9 × 15.9 × 15.9 nm^3^, respectively. The boxes were constructed such that the minimium distance between each BChE glycoform to the periodic boundary is 1.2 nm. Both BChE production simulations were performed for 100 ns in NPT with data collected every 0.1 ns. Each production run was preceded by an energy minimization in vacuum, solvation, solvated energy minimization, a 100 ps NVT equilibration, and subsequently 100 ps NPT equilibration. Both energy minimizations were terminated using a maximum force tolerance of 1000 kJ mol^-1^nm^-1^. In the solvation step, both systems were first neutralized with the addition of Na^+^ or Cl^-^ ions, and then concentrated to 155 mM NaCl. The Velocity–rescale thermostat [39] was used with a reference temperature of 310 K, using a time constant of 0.1 ps. Solvent molecules were thermostatted separately from the glycan and protein residues. The isotropic Parrinello–Rahman barostat [40] was used with a reference pressure of 1 bar, using a time constant of 2 ps and isothermal compressibility of 4.5 × 10^−5^ bar^−1^. Temperature, pressure, and NaCl concentration were selected to emulate human body conditions. Harmonic position restraints were applied to the protein atoms during the NVT and NPT equilibrations to ensure the equilibration process did not disrupt the natural protein fold. All nonbonded interactions employed a short–range cutoff of 1 nm, with vertically shifted potentials such that the potential at the cutoff range is zero. The Particle–Mesh Ewald method [41] with cubic interpolation was used to model long–range electrostatic interactions. Dispersion correction was applied to both energy and pressure.

## Results and discussion

**Fig 4:**
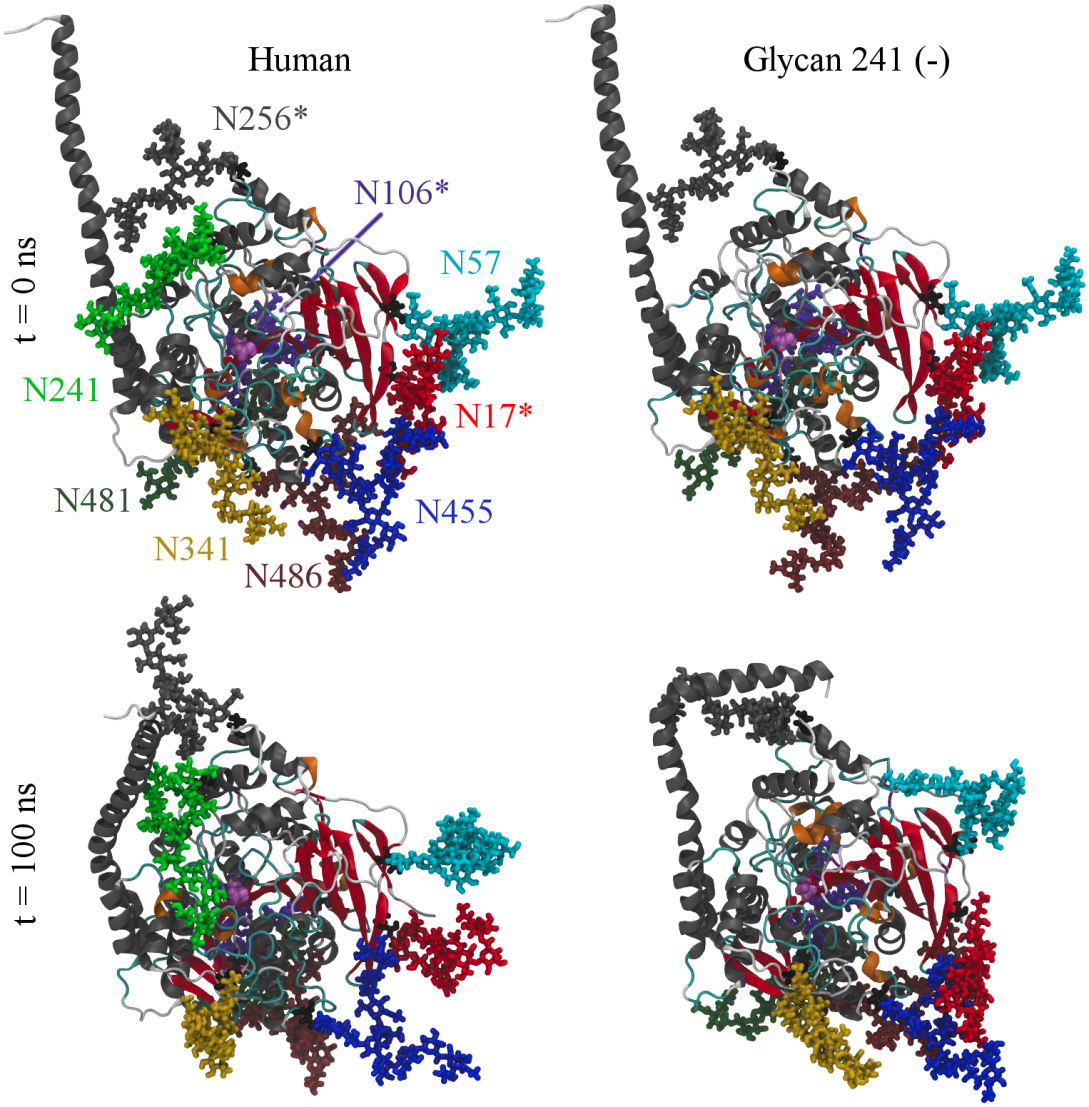
Initial and final conformations. Initial (top) and final (bottom) conformations of the simulated human (left) and glycan 241 (–) (right) glycoforms. Images are oriented such that the reader is viewing directly into the active site gorge. Glycans are labeled in the top left image by color. An asterisk denotes a monosialylated glycan. The active site SER_198_ is colored in pink.

BChE contains nine sites that can be glycosylated, as shown in Table 1. The attached glycans for both glycoforms in their initial and final conformations are shown in Fig 4. Note that the starting protein conformation for glycan 241 (–) simulation is the same as that shown in human at *t* = 0 ns, while the starting protein conformation of glycan 241 (+) simulation is identical to glycan 241 (–) at *t* = 100 ns. Videos of their stabilized trajectories are provided (see S1, S2 and S3 Movies). The coordinate and topology files for all simulations are included in S1 File.

### Protein backbone RMSD

**Fig 5:**
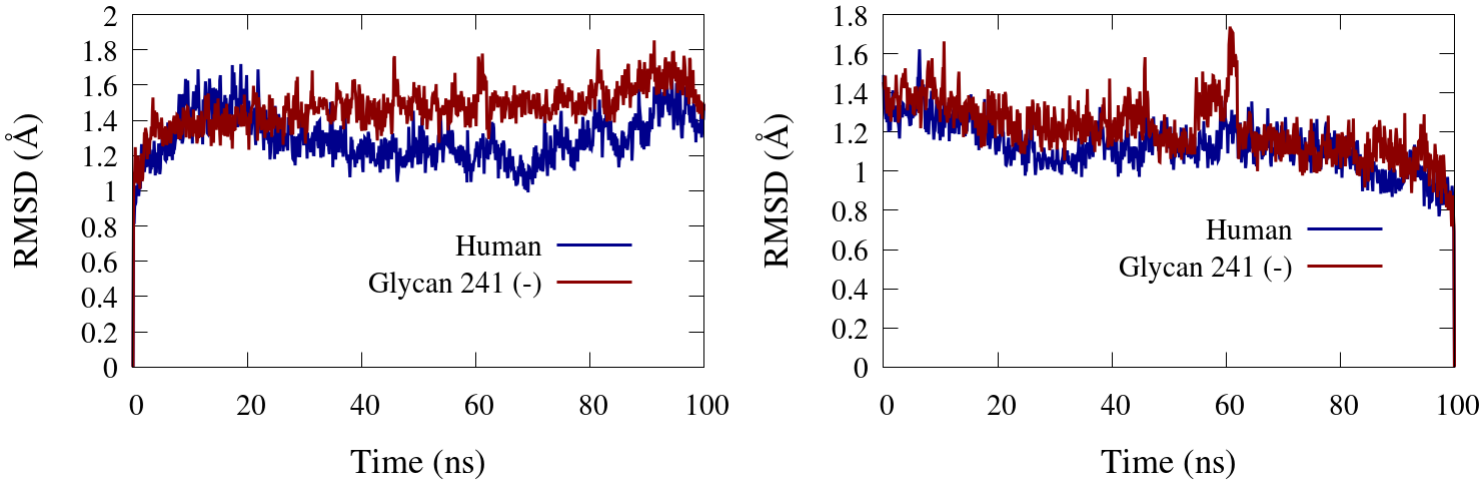
Core backbone RMSD. Core backbone residues (5–529) atomic RMSD over 100 ns from the initial conformation (left) and the final conformation (right) for the human and glycan 241 (-) simulations. of BChE.

Root–mean–square deviation (RMSD) of the backbone atoms of the core domain from the initial and final structures of the human and glycan 241 (–) are shown in Fig 5. The RMSD at time *t*_*i*_ = *i*Δ*t*, where Δ*t* is the time resolution of the data, was calculated according to Eq 1

$$\begin{array}{c} \text{RMSD}\left(t_{i}\right)=\sqrt{\frac{1}{N}\;\sum_{j=1}^{N}\;\Delta d_{ij}^{2}}, \end{array}$$ (1)

where *N* is the number of backbone atoms and Δ*d*_*ij*_ is the deviation of atom *j* at time *t*_*i*_ from a reference position. RMSD was calculated post removal of translational and rotational displacement of the protein’s core domain residues 5–529 with the GROMACS “gmx rms” module. Residues 1–4 and 530–574 were not included in the RMSD analysis since these residues constitute the flexible regions of the protein, and contribute a significant skew in the backbone RMSD values. The BChE glycoforms’ core backbone RMSD values were below two angstroms, confirming that the folded state was preserved throughout the simulation. We note that both the initial and final frame RMSDs generally display trending, indicating that our conformational sampling of BChE’s core backbone residues has not yet fully equilibrated. This is reasonable, as protein folding is known to take place on the order of *μ*s to s [42]. RMSD from the initial conformation reveals that the structure of the human glycoform’s core domain is significantly closer to its initial conformation than glycan 241 (–) from approximately 30 ns to 90 ns. RMSD from the final conformation shows that the final conformation of both glycoforms is generally equally deviant from the rest of its respective simulation, except for the glycan 241 (–) glycoform at around 60 ns, where we see a 0.2 Å spike in the RMSD. Interestingly, this spike is significantly less pronounced in the RMSD from the initial conformation, indicating that RMSD from the final conformation is a more sensitive measure of fold deviations through the simulation.

### Conformational dihedral angle analysis

**Fig 6:**
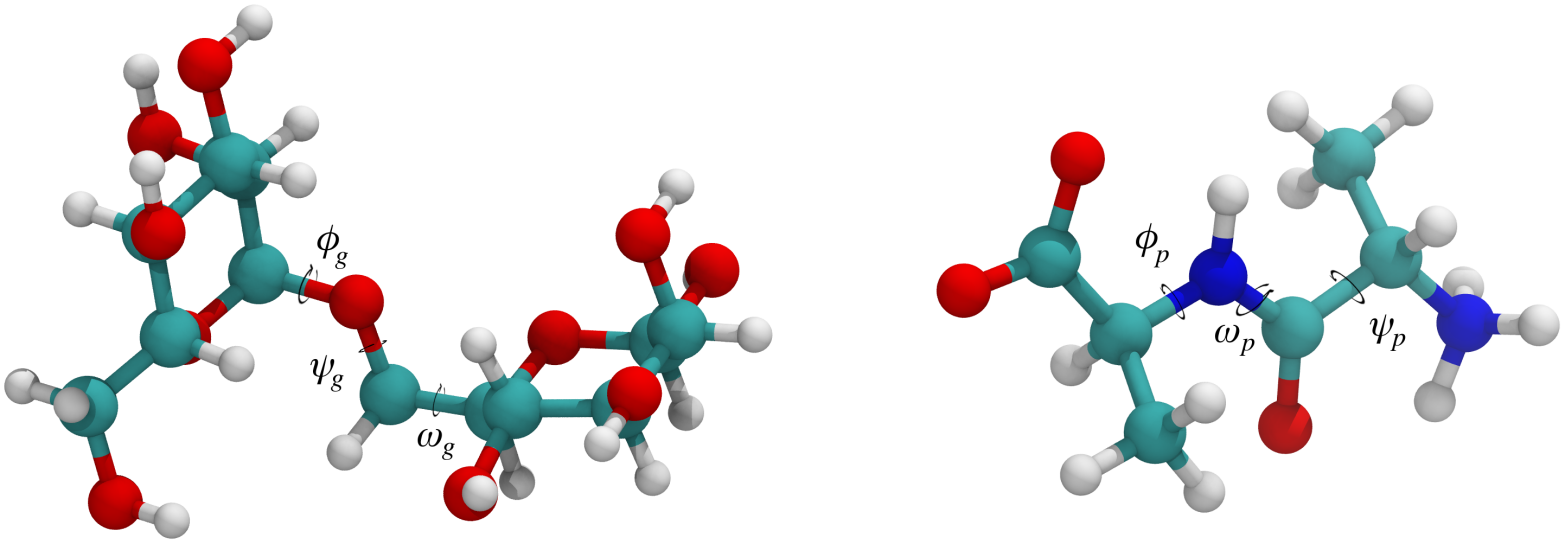
Glycan and protein conformational dihedral angles. Glycan conformational dihedral angles *ϕ*_*g*_, *ψ*_*g*_, and *ω*_*g*_ of *α*1-6 dimannose (left) and protein conformational dihedral angles *ϕ*_*p*_, *ψ*_*p*_, and *ω*_*p*_ of dialanine (right). Glycan linkages only contain an *ω*_*g*_ when the linkage occurs on the terminal carbon; otherwise, the linkage only contains *ϕ*_*g*_ and *ψ*_*g*_.

The dynamics of proteins and glycans can be analyzed through conformational dihedral angles. For both, protein and glycans, there are three dihedral angles of interest, termed *ϕ*, *ψ* and *ω*. Note that despite there being three dihedral angles identically named for protein and glycan dihedrals, they are completely independent. Fig 6 depicts the locations of the glycan and protein conformational dihedral angles.

We observe the median square angular displacement (MSAD), mean angular displacement (MAD) and angular autocorrelation function (AACF) for all conformational dihedral angles in our simulations. The MSAD for a time lag *τ*_*i*_ = *i*Δ*t* is given by Eq 2

$$\begin{array}{c} \text{MSAD}\left(\tau_{i}\right)=\underset{l\in \Theta ,k\in T_{i}}{\text{median}}\;[{\left(\sum_{j=k}^{k+i-1}\;\Delta \theta_{jl}\right)}^{2}], \end{array}$$ (2)

where *l* denotes the *l*^th^ conformational dihedral angle in the set of conformational dihedral angles Θ, and Δ*θ*_*jl*_ denotes the signed angular displacement of the *l*^th^ conformational dihedral angle from time *t*_*j*_ to time *t*_*j*_ + Δ*t*. *T*_*i*_ is the set of usable time indices, which is simply all time indices included in simulation time *t* = 0 to *t* = *T* − *τ*_*i*_, where *T* is the total simulation time. The median was selected as a measure of central tendency as opposed to the arithmetic mean, since squared angular displacement follows a *χ*^2^ distribution, as angular displacement is Gaussian. This expression is valid as long as |Δ*θ*_*jl*_| ≤ 180° for all *j*, *l*, which is consistent with our time resolution Δ*t*.

The mean angular displacement (MAD) at a time lag *τ*_*i*_ is given by Eq 3

$$\begin{array}{c} \text{MAD}\left(\tau_{i}\right)=\frac{1}{N_{\theta }\left(N_{t}-i\right)}\;\sum_{l=1}^{N_{\theta }}\;\sum_{k=1}^{N_{t}-i}\;\sum_{j=k}^{k+i-1}\;\Delta \theta_{jl}, \end{array}$$ (3)

where *N*_*θ*_ is the number of conformational dihedral angles and *N*_*t*_ is the total number of time indices. The angular autocorrelation function (AACF) at time lag *τ*_*i*_ was calculated according to Eq 4 [43]

$$\begin{array}{c} \text{AACF}\left(\tau_{i}\right)=\underset{l\in \Theta ,k\in T_{i}}{\text{median}}\;[\cos\;(\sum_{j=k}^{k+i-1}\Delta \theta_{jl})]. \end{array}$$ (4)

**Fig 7:**
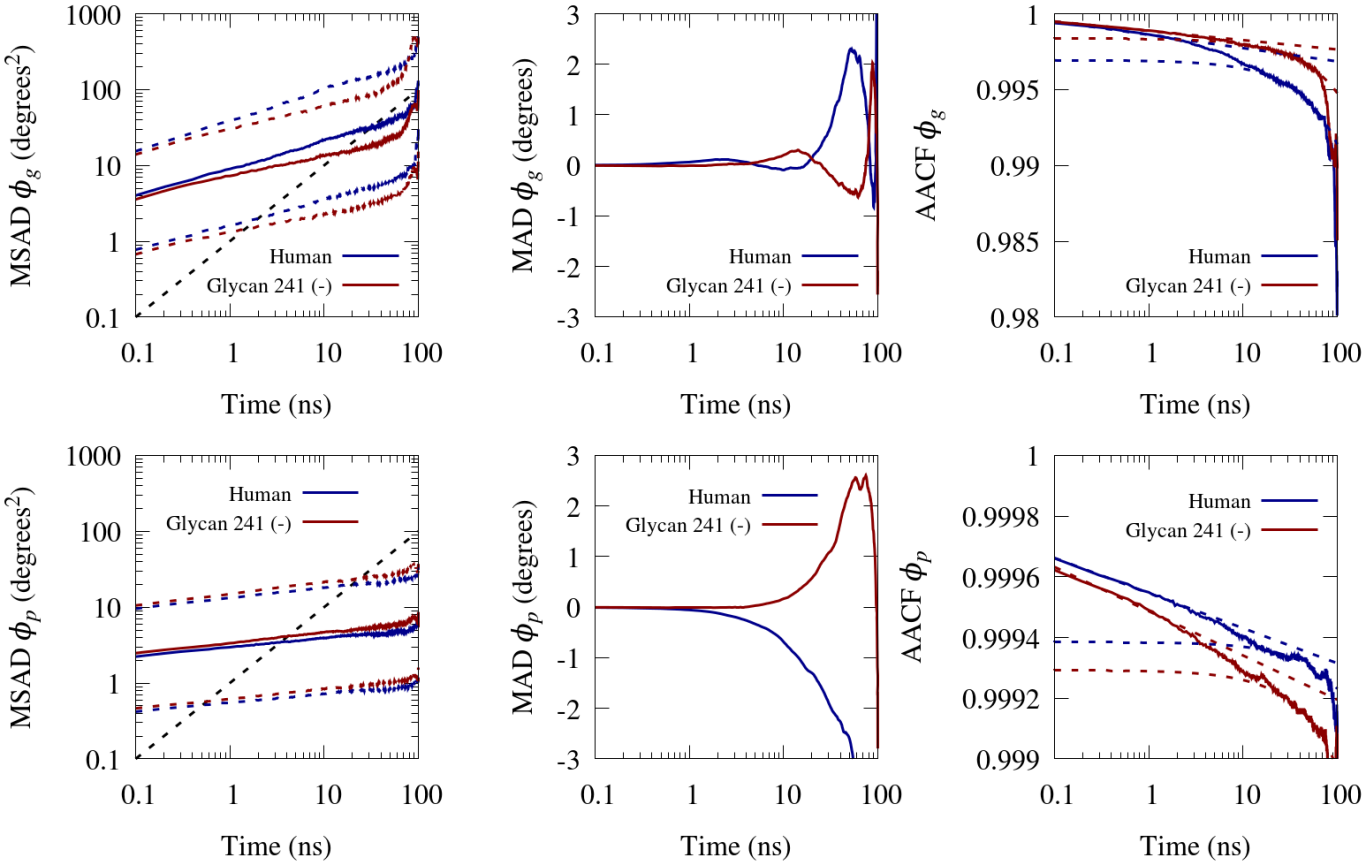
Dynamics of conformational dihedral angle *ϕ*_*g*_. MSAD (left column), MAD (middle column) and AACF (right column) of the *ϕ*_*g*_ (top row) and *ϕ*_*p*_ (bottom row) of the human and glycan 241 (–) BChE glycoform. MSAD is displayed in log–log plots, while MAD and AACF are displayed in semi–log plots. The first and third quartiles of the MSAD are shown in colored dashed lines, and a representative diffusive line is shown in a black dashed line. A power law fit from *τ* = 0–2 ns of the AACF is shown in a dashed line, and a linear fit from *τ* = 20–60 ns is also shown in a dashed line.

This AACF is the normalized autocorrelation function of the two vectors formed by projecting the two dihedral planes onto the orthogonal plane. Fig 7 shows the MSAD, MAD and AACF for the *ϕ* dihedral angles of the glycans in the human and glycan 241 (–) glycoforms. We see that there is not much difference between the glycan conformational dihedral angles in terms of MSAD, MAD, or AACF. The MSAD has a slope that is less than unity on a log–log plot, indicating sub–diffusive behavior. The AACF remains near unity and slowly decreases, indicating highly constrained dihedral motion. Comparing the MSAD, MAD and AACF of glycan conformational dihedral angles to protein conformational dihedral angles, the protein dihedral angles have a much flatter MSAD, indicating a greater degree of sub–diffusive behavior. The MAD appears to become unstable at around *τ* = 2 ns for both the protein and glycan conformational dihedral angles, but the behavior of the MAD instability between protein and glycan is clearly different. The AACF is approximated well by a power law decay during *τ* = 0–2 ns, which is consistent with previously reported power law autocorrelations in proteins [44, 45]. The AACF is better approximated by a linear function on the range of *τ* = 20–60 ns, indicating that our correlations have not equilibrated on this timescale. The AACF is a full order of magnitude closer to unity for the protein dihedral angles (1 − *O*(10^−4^) versus 1 − *O*(10^−3^)), indicating a higher degree of entrapment. This is expected as proteins are generally less flexible than glycans. We note that for a truly entrapped dihedral, MSAD would eventually level out, MAD would have a longer stability window, and AACF would approach a limiting value. This indicates that longer simulation timescales are required to reach equilibrium. Still, our simulations are long enough to provide dynamic insight on the 0.1 to 2 ns timescale.

**Fig 8:**
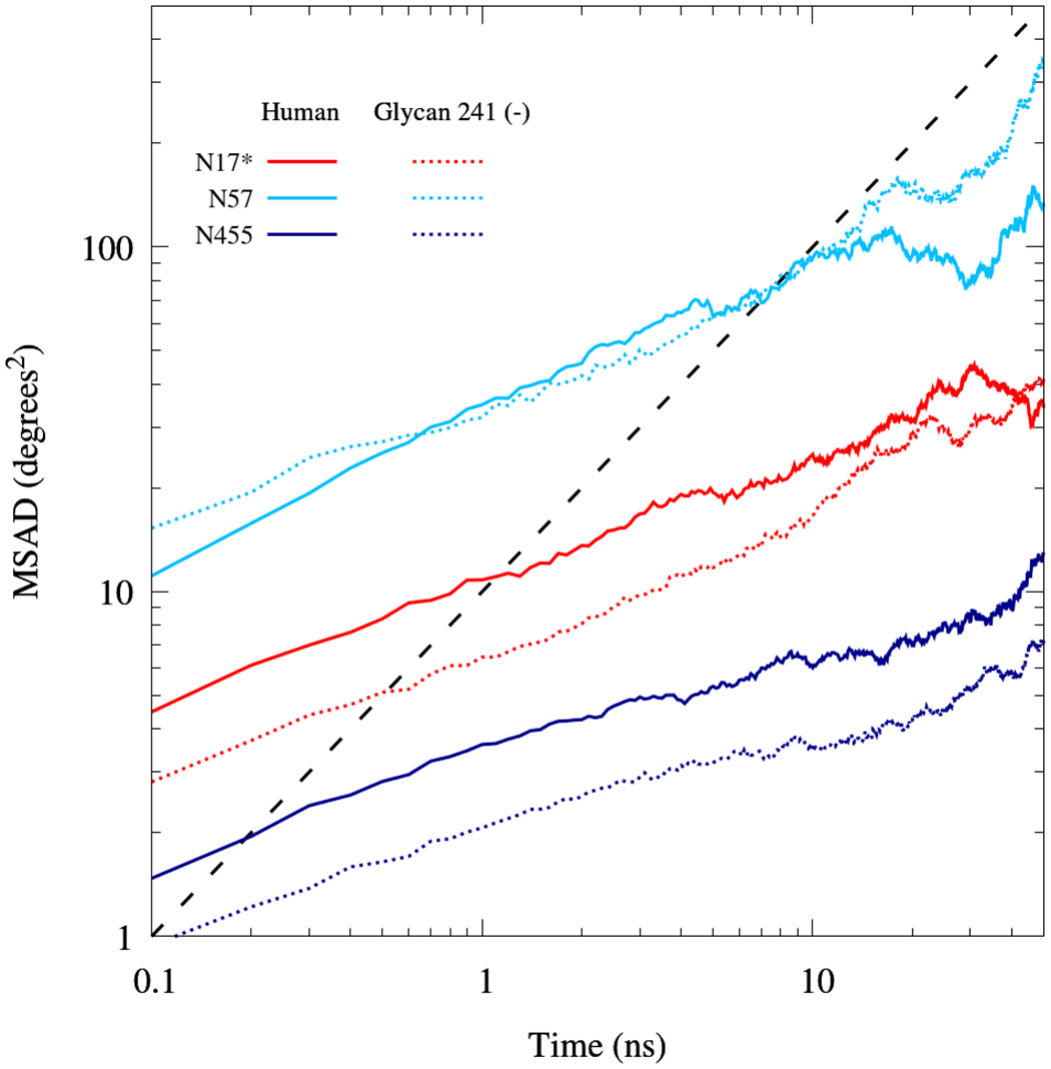
Representative glycan MSAD. MSAD of three representative glycans combining all *ϕ*, *ψ* and *ω* conformational dihedral angles in the Human and Glycan (–) glycoforms. Glycan 455 represents a lower bound, 57 represents a higher bound, and 17 represent a central glycan. The asterisk on Glycan 17 indicates a monosialilated glycan.

We are able to extract diffusivity information from the MSAD using the equation for one–dimensional anomalous diffusion given by Eq 5 [46]

$$\begin{array}{c} \text{MSAD}=2D_{\alpha }\tau^{\alpha }, \end{array}$$ (5)

where *α* is the anomalous diffusion exponent (*α* = 1 is diffusive, *α* < 1 is sub–diffusive, and *α* > 1 is super–diffusive) and *D*_*α*_ is the prefactor (which for *α* = 1 becomes the diffusion coefficient). Fig 8 shows the combined MSAD for three representative glycans (see S3 Fig for the complete plot). The glycan MSAD has roughly an order of magnitude spread for both glycoforms. The anomalous diffusion parameters for glycan conformational dihedral angles combined and categorized by glycan are shown in Table 2. We note that the glycan 241 (–) glycan has higher *α* and *D*_*α*_ for the following 6 of its 8 glycans: 17, 57, 106, 455, 481 and 486. The reduced crowding resulting from the absence of glycan 241 is likely the cause of the increased mobility of these glycans.

**Table 2:** Anomalous diffusion parameters *α* and *D*_*α*_ for all glycan conformational dihedral angles categorized by glycan.

Glycoform:	Human		Glycan 241 (–)	
Glycan	*α*	*D*_*α*_ (deg.^2^/ ns^*α*^)	*α*	*D*_*α*_ (deg.^2^/ ns^*α*^)
17*	0.39	3.22	0.35	5.40
57	0.40	16.71	0.42	17.05
106*	0.15	1.72	0.47	2.48
241	0.32	4.88	–	–
256*	0.31	7.46	0.27	7.75
341	0.27	1.24	0.30	1.19
455	0.27	1.03	0.28	1.71
481	0.18	6.53	0.39	6.20
486	0.26	4.74	0.58	8.82

*monosialylated glycan

The parameters extracted from the glycan and protein MSAD are shown in Table 3. For both glycoforms, *ω*_*g*_ has greater *α* and *D*_*α*_ than both *ϕ*_*g*_ and *ψ*_*g*_, which is to be expected since *ω*_*g*_ is always adjacent to two other fully rotatable bond, increasing its relative rotational freedom. The glycan conformational dihedral angle *α* and *D*_*α*_ are significantly greater than the protein conformational dihedral angles, consistent with glycans being more flexible than proteins. The *α* and *D*_*α*_ for the protein dihedral angles are consistently higher for the human glycoform than the glycan 241 (–) glycoform. This may be attributed to a reduction in mobility of the tetramerization domain for the glycan 241 (–) glycoform. This trend is directly related to the glycan conformational mobility, as the glycan 241 (–) has consistently higher *α* and *D*_*α*_ for the glycan conformational dihedral angles.

**Table 3:** Anomalous diffusion parameters *α* and *D*_*α*_ for all glycan and protein conformational dihedral angles categorized by conformational dihedral angle.

Glycoform:	Human		Glycan 241 (−)	
Angle	*α*	*D*_*α*_ (deg.^2^/ ns^*α*^)	*α*	*D*_*α*_ (deg.^2^/ ns^*α*^)
*ϕ*_*g*_	0.27	3.60	0.37	4.47
*ψ*_*g*_	0.28	3.21	0.38	4.28
*ω*_*g*_	0.33	4.34	0.40	5.87
*ϕ*_*p*_	0.14	1.69	0.12	1.48
*ψ*_*p*_	0.14	1.71	0.12	1.50
*ω*_*p*_	0.14	1.68	0.12	1.52

Glycan conformational dihedral angles are denoted by the *g* subscript and protein are denoted by the *p* subscript.

### BChE active site

**Fig 9:**
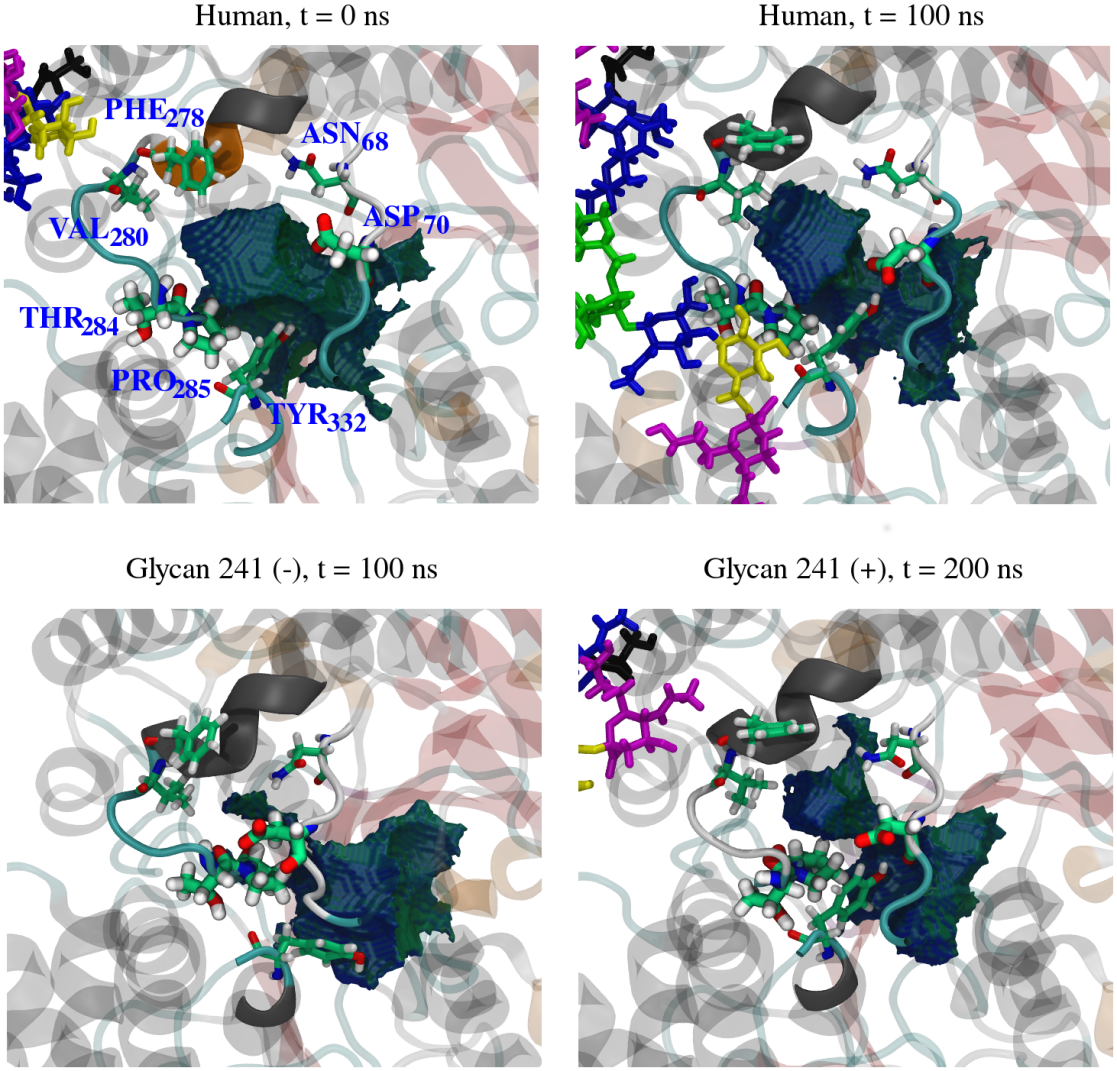
Active site gorge. Active site cavity images of the simulated BChE glycoforms. The human cavity at *t* = 0 ns (top left) is representative of the glycan 241 (–) cavity at *t* = 0 ns. The glycan 241 (–) cavity at *t* = 100 ns (bottom left) is representative of the glycan 241 (+) cavity at *t* = 100 ns. Cavities were generated using VOIDOO [18]. The cavity surface shown represents the surface accessible to the center of a probe of radius 1.4 Å. Protein residues lining the entrance are opaque, with select residues in licorice representation. ASP_70_, THR_284_ and PRO_285_ are enlarged, as their grouped center of mass distance distance is used to represent gorge accessibility. Glycan 241 is the only glycan shown, and is colored according to Fig 3.

**Fig 10:**
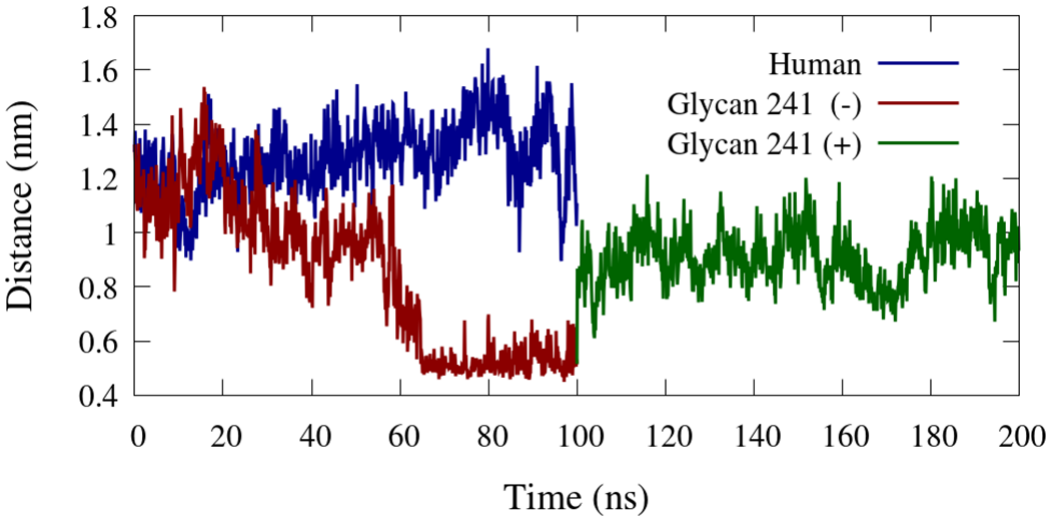
Active site residue distance. Center of mass distance between ASP_70_ and combined residues THR_284_ and PRO_285_. These residues are located at the top of the active site gorge, and characterize the opening and closing of the gorge.

The active site conformation of BChE was affected by the presence or absence of glycan 241. Fig 9 depicts the starting and ending conformations of the active site gorge for representative simulated BChE glycoforms. The initial and final cavity volumes of the gorge are provided in S1 Table. The accessibility of the gorge was found to be directly related to the center of mass distance between residues ASP_70_ and combined residues THR_284_ and PRO_285_, displayed in Fig 10. The gorge remains accessible throughout the simulation for the human glycoform. However, the glycan 241 (–) glycoform’s gorge becomes inaccessible at 65 ns, when the distance for the glycan 241 (–) glycoform becomes and remains around 0.5 nm. The active site closure event occurs directly proceeding the RMSD spike at 60 ns, exhibited in Fig 5, right. The human glycoform simulation did not exhibit any closure events through its trajectory. In order to affirm the correlation between the active site closure and the presence of glycan 241, an additional simulation was performed on the fully glycosylated form whose initial coordinates were taken from the glycan 241 (–) at 100 ns (i.e. glycan 241 (+)). From Figs 9 and 10, we see the gorge quickly reopens upon reintroduction of glycan 241. We note that the distance in Fig 10 for the glycan 241 (+) does not reach the value of the human glycoform simulation. Fig 9 top right shows the *α* 1,3–arm galactose and N–-Acetylneuraminic acid in close proximity to gorge–lining residues. This interaction is not present in the glycan 241 (+) simulation, likely due to limited simulation time. Collectively, this information suggests that glycan 241 is an important factor in the activity of BChE glycoforms.

### BChE tetramerization domain

**Fig 11:**
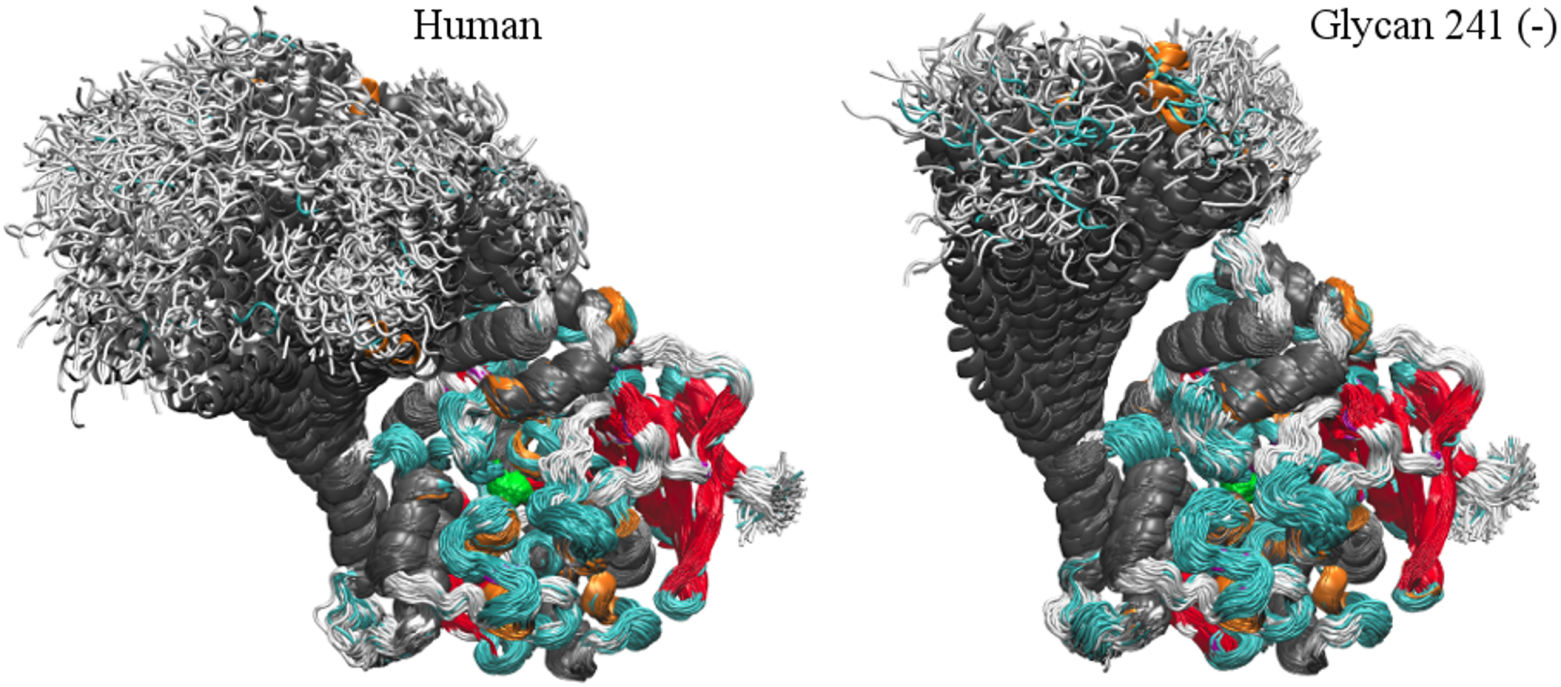
Secondary structure overlays. 100 ns, 1000 frame secondary structure overlays of human (left) and glycan 241 (–) (right) BChE glycoforms. *α*–helices are colored in gray, *β*–sheets in red, 3_10_–helices in orange, coil in white and turn in cyan. The active site SER_198_ is colored in green.

The behavior of the tetramerization domain was highly dependent on the simulated BChE glycoform. Fig 11 shows secondary structure overlays of the simulated BChE glycoforms, omitting the glycans. The active site SER_198_ is occluded in the glycan 241 (–) simulation, indicating that the glycan attached to ASN_241_ is critical to BChE activity.

**Fig 12:**
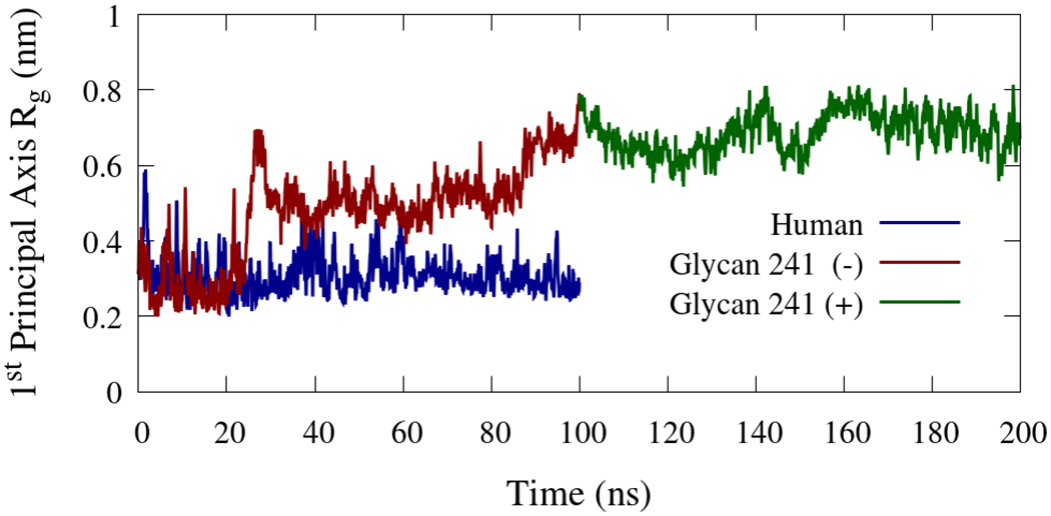
Tetramerization domain radius of gyration. Radius of gyration along the longest principal component of the BChE tetramerization domain, residues 530–574.

The shape of the BChE tetramerization domain may be characterized by the radius of gyration *R*_*g*_ along its first principle component. The *R*_*g*_ of the BChE tetramerization domain for the human and glycan 241 (–) glycoforms are displayed in Fig 12, calculated using the GROMACS “gmx gyrate” module. This statistic describes how wide the tetramerization domain is perpendicular to its longest axis. The glycan 241 (–) glycoform has a distinctly wider *R*_*g*_ distribution after 100 ns of simulation than the human glycoform, consistent with the visible bend in the tetramerization domain, exhibited in Fig 4, bottom right. From Fig 12, the tetramerization domain of the glycan 241 (–) glycoform becomes locked into its bent conformation around 28 ns, while the human glycoform remains generally elongated through the entire simulation. The glycan 241 (+) simulation also retains a bent tetramerization domain, possibly trapped in a local minimum energy structure (see S1, S2 and S3 Movies). We hypothesize that an extension of the glycan 241 (+) simulation would result in a conformation closer to that of the human simulation.

## Conclusion

The human BChE glycoform was simulated with and without glycan 241 present. Fully glycosylated human BChE was generally more structurally stable, exhibiting lower RMSD and lower mobility in glycan conformational dihedral angles. From the MAD and AACF data we found that 100 ns simulations were long enough to provide dynamical insight on conformational dihedral angles of glycans and protein for *τ* = 0–2 ns. We note that our conformational dihedral angle data indicates that longer simulation times are required for an accurate analysis of the equilibrium conformational ensemble of BChE, which is especially true for the highly flexible glycans. Nevertheless, useful structural information is obtained from the timescale simulated within this work. Our simulations indicate that the activity of the BChE monomer is heavily dependent on the presence of glycan 241. This dependency was exhibited by the distance measurement of ASP_70_ with combined residues THR_284_ and PRO_285_ and correlated to the RMSD from the final conformation. The closed conformation of the glycan 241 (–) glycoform was resimulated with glycan 241 reintroduced, and the active site partially reopened. The tetramerization domain remained bent in the glycan 241 (+) simulation. If the tetramerization domain in the glycan 241 (+) simulation were elongated as it is in the tetramer, we hypothesize that the active site will eventually fully open.

## Supporting information

S1 Table Initial and final cavity volumes computed by VOIDOO. (PDF)

S1 Fig Representative dihedral probability distribution. Representative dihedral angle probability distribution for GlcNac using Amber (blue), Gromacs with the original ACPYPE (red), and Gromacs with a modified version of ACPYPE that correctly transfers 1–4 scaling parameters (green).
(TIF)

S2 Fig Rotable bonds for glycan energy minimization. N–glycosidic and *ω*_*p*_ bonds for which the glycans are rotated along for the initial energy minimization procedure.
(TIF)

S3 Fig Glycan MSAD. MSAD of all glycans combining all *ϕ*_*g*_, *ψ*_*g*_, and *ω*_*g*_ conformational dihedral angles in the Human and Glycan (–) glycoforms. The color scheme corresponds directly to the glycans represented in Fig 4.
(TIF)

S1 Movie Video of the 100 ns human BChE simulation. Solvent omitted and glycoform stabilized by core residues 5–529.
(MP4)

S2 Movie Video of the 100 ns glycan 241 (–) BChE simulation. Solvent omitted and glycoform stabilized by core residues 5–529.
(MP4)

S3 Movie Video of the 100 ns glycan 241 (+) BChE simulation. Solvent omitted and glycoform stabilized by core residues 5–529.
(MP4)

S1 File Simulation files. Starting coordinate and topology files for the simulations presented in this work in GROMACS format. These files are post–glycan energy minimization.
(ZIP)
