# Supplementary material for: Structural analysis of human glycoprotein butyrylcholinesterase using atomistic molecular dynamics: The importance of glycosylation site ASN241
Source: PLoS One. 2017 Nov 30;12(11):e0187994. doi: 10.1371/journal.pone.0187994 (PMC5708630; doi:10.1371/journal.pone.0187994)
Supplement: S1 Table — (PDF) [file pone.0187994.s001.pdf]

Glycoform	$V_{initial}$ (Å^3)	V_{final} (Å^3)
Human	467	399
Glycan 241 (−)	467	387
Glycan 241 (+)	387	379

Table S1: Initial and final cavity volumes computed by VOIDOO.
