# Supplementary figures and images for: Structural analysis of human glycoprotein butyrylcholinesterase using atomistic molecular dynamics: The importance of glycosylation site ASN241
Source: PLoS One. 2017 Nov 30;12(11):e0187994. doi: 10.1371/journal.pone.0187994 (PMC5708630; doi:10.1371/journal.pone.0187994)

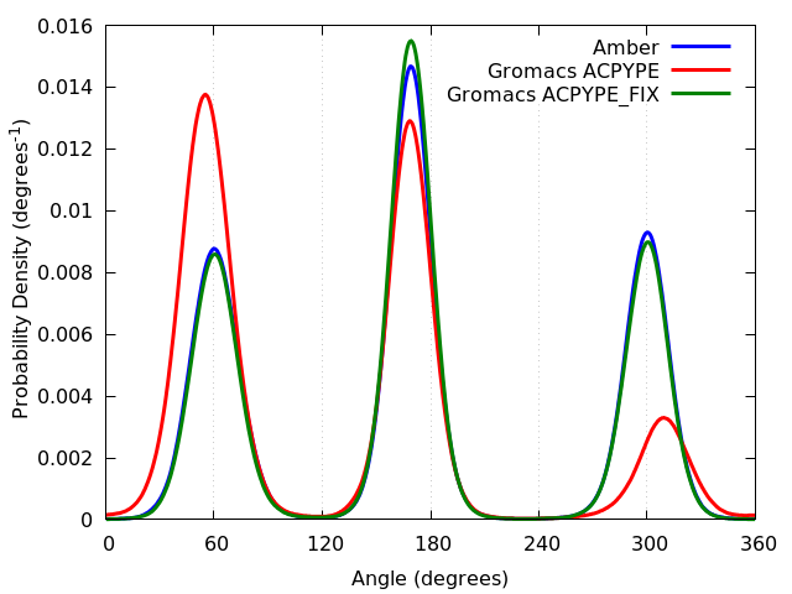

Supplement: S1 Fig — Representative dihedral angle probability distribution for GlcNac using Amber (blue), Gromacs with the original ACPYPE (red), and Gromacs with a modified version of ACPYPE that correctly transfers 1–4 scaling parameters (green). (TIF) [file pone.0187994.s002.tif]

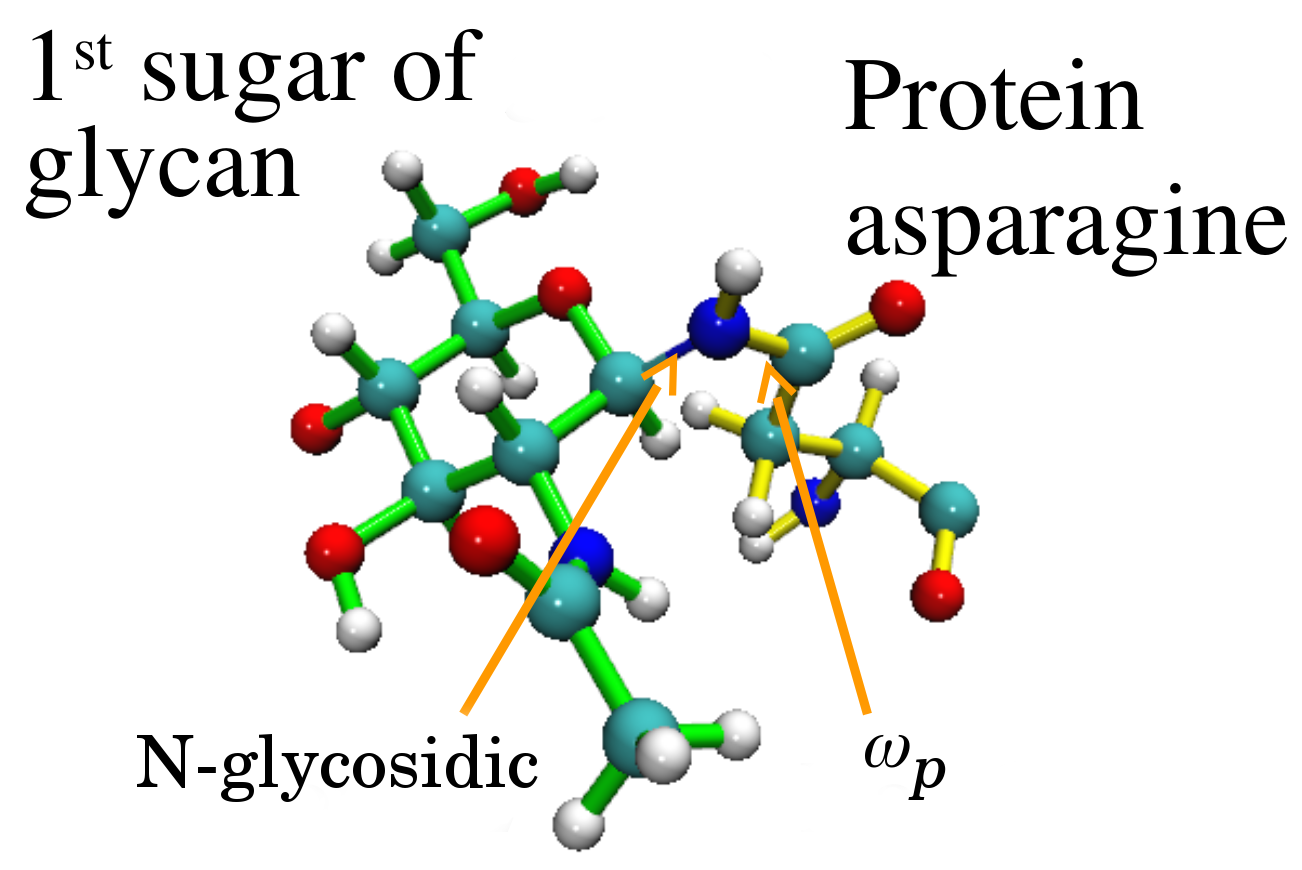

Supplement: S2 Fig — N–glycosidic and ωp bonds for which the glycans are rotated along for the initial energy minimization procedure. (TIF) [file pone.0187994.s003.tif]

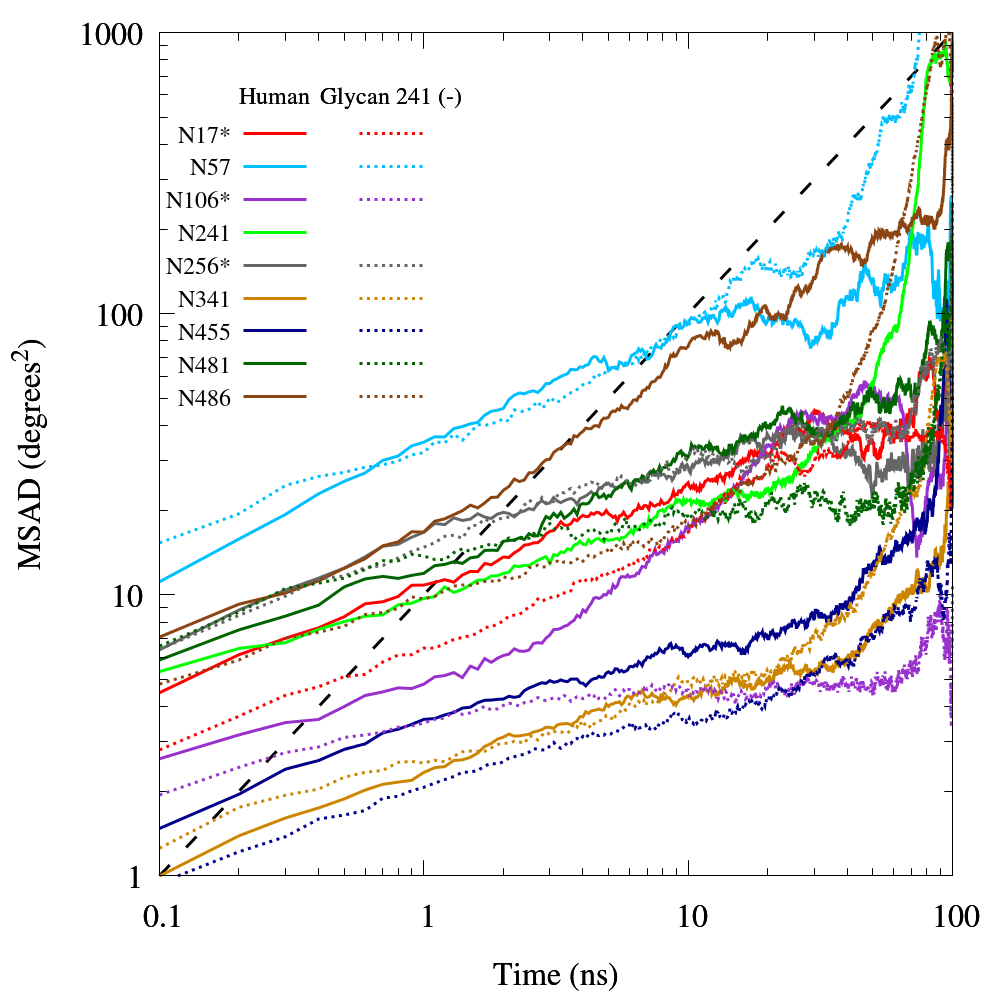

Supplement: S3 Fig — MSAD of all glycans combining all ϕg, ψg, and ωg conformational dihedral angles in the Human and Glycan (–) glycoforms. The color scheme corresponds directly to the glycans represented in Fig 4. (TIF) [file pone.0187994.s004.tif]
